# Attention-Based 3D Human Pose Sequence Refinement Network

**DOI:** 10.3390/s21134572

**Published:** 2021-07-03

**Authors:** Do-Yeop Kim, Ju-Yong Chang

**Affiliations:** Department of Electronics and Communication Engineering, Kwangwoon University, Seoul 01897, Korea; dyub1@kw.ac.kr

**Keywords:** 3D human mesh reconstruction, 3D human pose estimation, deep neural network

## Abstract

Three-dimensional human mesh reconstruction from a single video has made much progress in recent years due to the advances in deep learning. However, previous methods still often reconstruct temporally noisy pose and mesh sequences given in-the-wild video data. To address this problem, we propose a human pose refinement network (HPR-Net) based on a non-local attention mechanism. The pipeline of the proposed framework consists of a weight-regression module, a weighted-averaging module, and a skinned multi-person linear (SMPL) module. First, the weight-regression module creates pose affinity weights from a 3D human pose sequence represented in a unit quaternion form. Next, the weighted-averaging module generates a refined 3D pose sequence by performing temporal weighted averaging using the generated affinity weights. Finally, the refined pose sequence is converted into a human mesh sequence using the SMPL module. HPR-Net is a simple but effective post-processing network that can substantially improve the accuracy and temporal smoothness of 3D human mesh sequences obtained from an input video by existing human mesh reconstruction methods. Our experiments show that the noisy results of the existing methods are consistently improved using the proposed method on various real datasets. Notably, our proposed method reduces the pose and acceleration errors of VIBE, the existing state-of-the-art human mesh reconstruction method, by 1.4% and 66.5%, respectively, on the 3DPW dataset.

## 1. Introduction

Three-dimensional human pose estimation is an important and actively studied problem in computer vision. Various methods have been proposed to generate successful pose estimation results on the basis of deep learning. These methods have been used to address the problem of reconstructing 3D human pose from a single RGB image or video obtained from a monocular camera. Recently, methods for estimating dense 3D mesh beyond sparse 3D joints have been proposed on the basis of a statistical shape model for human body. However, reconstructing 3D human poses from RGB images accurately remains a difficult problem.

**Figure 1:**
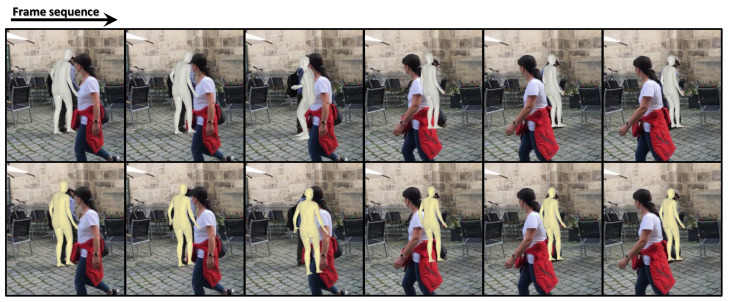
This figure shows a 3D human mesh sequence estimated by VIBE (**top row**) and its refined result by our proposed method (**bottom row**). In the 3rd frame, VIBE fails to estimate the correct pose of the target person due to severe occlusion. Our method effectively refines the incorrectly estimated results.

Recent methods for 3D human mesh reconstruction extract features from input images on the basis of deep learning and directly regress the pose and identity parameters of a statistical shape model, such as a skinned multi-person linear model (SMPL) [1], from the extracted features. However, in the case of an image including occlusion or an unseen pose that is not included in training data, the network has difficulty in estimating the correct pose. Methods of estimating temporally coherent pose sequences from input videos have shown moderate performance [2,3,4]. However, the above problems still prevent the existing methods to reconstruct the correct pose in some frames and generate noisy human motion. For example, the top row of Figure 1 shows the results obtained by VIBE [3], a state-of-the-art method for reconstructing 3D human mesh from video. In the 3rd frame, VIBE fails to estimate the correct pose, resulting in temporally noisy results. Our study focusses on this problem, namely temporally coherent human pose estimation from input video. Specifically, we propose a *human pose refinement network* (i.e., HPR-Net) that can refine the noisy human pose sequence reconstructed by existing methods. The bottom row of Figure 1 shows the improved results through our HPR-Net.

Weighted averaging is a simple but effective method that has been widely used for refinement of signals, including images. The basic idea of this paper to refine the noisy 3D human pose sequence is based on weighted averaging. However, applying weighted averaging to human pose refinement is not trivial due to the following two problems. The first problem is how to determine weights for weighted averaging. To accomplish this task, we learn a module that generates optimal weights on the basis of large-scale data. Specifically, we define a weight as affinity between two 3D poses. We propose a non-local attention-based weight regression module that can consider long-range interactions to compute this affinity. The proposed module is supervised to output weights that can reconstruct a ground-truth pose sequence from a noisy pose sequence estimated by existing human pose estimation methods.

Our human pose refinement method relies on SMPL, where 3D human pose is represented as a set of 3D rotations of joints. However, 3D rotation and 3D pose, including the rotation, cannot be regarded as a vector defined in Euclidean space. Thus, weighted averaging cannot be applied directly to 3D human poses. Performing weighted averaging for 3D rotation requires a complex optimization process [5]. To alleviate this problem, we use Gramkow’s study [6], which proves that the mean of unit quaternions is a quadratic approximation of the mean of 3D rotations. Specifically, we first represent the 3D rotation constituting the 3D human pose as a unit quaternion and then perform weighted averaging on the 3D human pose sequence represented as a sequence of unit quaternions. This weighted averaging based on the unit quaternion can be represented as a simple algebraic equation without an optimization process and can be included in our network for learning.

**Figure 2:**
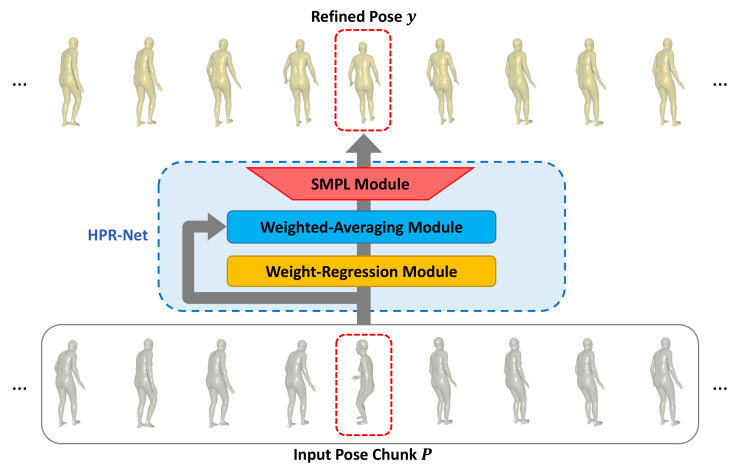
Overall framework of the proposed method. The input to our model is a noisy 3D human pose sequence estimated by existing 3D human pose estimation methods. Our proposed HPR-Net refines the noisy 3D human pose sequence and generates a refined human pose sequence.

Suppose that SMPL-based human mesh reconstruction methods estimate the 3D pose and identity parameter sequence. Our proposed system consisting of a weight-regression module, a weighted-averaging module, and an SMPL module performs pose refinement for a noisy 3D pose sequence through the following process. To refine a pose of a frame, the weight-regression module first generates weights for the poses in a window of a predefined size around that frame. Next, the weighted-averaging module outputs an improved 3D pose by applying weighted averaging on the basis of the generated weights to the poses inside the window. Finally, the SMPL module generates human meshes and 3D joints from the improved 3D pose parameters. This process is repeated for all frames to reconstruct the refined 3D pose and mesh sequence. An overview of the proposed method is shown in Figure 2.

The contributions of this paper can be summarized as follows:

- We propose a novel method to refine a 3D human pose sequence consisting of 3D rotations of joints. The proposed method performs human pose refinement independently from existing 3D human pose estimation methods. It can be applied to the results of any existing method in a model-agnostic manner and is easy to use.
- The proposed method is based on a simple but effective weighted-averaging operation and generates interpretable affinity weights using a non-local attention mechanism.
- In accordance with our experimental results, the proposed method consistently improves the 3D pose estimation and mesh reconstruction performance (i.e., accuracy and smoothness of output sequences) of existing methods for various real datasets.

## 2. Related Work

**Human mesh reconstruction.** Many recent 3D human mesh reconstruction methods directly regress the parameters of statistical shape models, such as SMPL [1]. These methods can be broadly classified into a single image-based approach [7,8,9,10] and a video-based approach [2,3,4].

The single image-based approaches reconstruct 3D human mesh from a monocular image. Bogo et al. [7] proposed a method that estimates 2D joints from an input image on the basis of a pretrained 2D joint regression network and optimizes an energy function to fit SMPL to the regressed 2D joints. Pavlakos et al. [8] extended [7] to optimize an improved energy function to fit the SMPL-X model to the regressed 2D full-body joints for holistic body modeling. A variational autoencoder(VAE)-based pose prior for valid pose parameter regression was proposed for optimization. Kanazawa et al. [9] proposed a model that directly maps features extracted by a deep network from a single image to SMPL parameters. In their method, an adversarial prior for the estimated parameters was proposed and learned to help obtain a realistic human mesh. Kolotouros et al. [10] combined the optimization-based method and the regression-based method in an end-to-end manner. The SMPL parameter estimated from a single image is used as an initial parameter, which is iteratively optimized through the method of [7]. The optimized parameter is used as a pseudo ground-truth for learning the regressor to construct a self-improving framework.

The video-based approaches reconstruct the 3D human mesh sequence from a video. Kanazawa et al. [2] proposed a temporal convolutional network that reconstructs the SMPL model from an image sequence. This method is supervised to predict SMPL models in the nearby few frames to learn information about human motion better. It can estimate past and future meshes from a single image through a hallucinator. Kocabas et al. [3] proposed a method that reconstructs an SMPL model sequence from a feature sequence computed using bidirectional gated recurrent unit from an input video. To compensate for the lack of 3D annotated data, this method performs weak supervision with various 2D datasets and adversarial training using large-scale motion datasets, resulting in successful human mesh reconstruction performance. Luo et al. [4] tried to solve the jittering problem from the inference results of existing methods for video data. This method reconstructs coarse motion by learning a VAE-based motion prior and then performs refinement for each frame’s pose. Thus, the smoothness of the output SMPL sequence is improved. Despite these recent advances in 3D human mesh reconstruction, most methods still produce erroneous poses or jittered motions due to unseen poses or occlusions from input images or videos acquired in an uncontrolled environment. Our work can substantially improve the accuracy and smoothness of human mesh sequences reconstructed by existing methods.

**Non-local attention.** Non-local attention was proposed to model long-range dependency in natural language processing [11,12] and computer vision [13,14,15]. Vaswani et al. [12] proposed the transformer, which is a framework using only attention mechanisms to overcome the limitations of existing recurrent models for natural language processing tasks and successfully solves the long-range dependency problem. Recently, the transformer architecture is known to improve image recognition performance and is actively used for various computer vision tasks [16,17,18,19,20]. Wang et al. [13] attempted to model the long-range dependency in image features using non-local operations proposed in [21]. For this, a non-local block based on attention mechanisms was proposed. On this basis, the method in [12] can be regarded as a special case of non-local neural networks. In the study of Cao et al. [14], the position-wise attention map of [13] was analyzed qualitatively, and most of the attention maps of each position have similar attention aspects. On this Basis, a more efficient non-local attention block was proposed. Woo et al. [15] proposed a method that extracts new features by successively applying channel attention and spatial attention to input features. This method shows a stronger representation power than features based on existing fully convolutional baselines. Our method generates a temporal non-local attention map inspired by [13,21]. The generated attention weights suppress features that are useless for refinement and strengthen helpful features. Our method can refine noisy pose parameter sequences through this attention mechanism.

**Human pose refinement.** The goal of human pose refinement studies is mainly to refine an estimated sparse joint set. Existing pose refinement methods are included as part of the joint regression network or used as a post-processing module for inference results. Newell et al. [22] proposed a network in which several hourglass modules are stacked. Hourglass module repeats top-down and bottom-up processing, extracts features at various scales, and is trained with intermediate supervision. Each stage module generates a heatmap, which is used as input to the next stage module for refinement. Chen et al. [23] proposed a cascaded pyramid network that combines GlobalNet, a Resnet-based pyramid network, and RefineNet, which refines the heatmap generated by GlobalNet. RefineNet considers all features obtained from each step of the pyramid to find occluded joint positions that are difficult to estimate. Moon et al. [24] proposed a model-agnostic refinement model based on the error distribution of 2D pose estimation models investigated in Ronchi et al.’s work [25]. This method is independent on the pose estimation model because it does not work in an end-to-end manner, and pose estimation performance can be improved for various existing approaches. Mall et al. [26] proposed a method to refine noisy motion capture data. The proposed network consisting of linear layers and bidirectional long short-term memories regresses the standard deviation of a Gaussian kernel to improve the pose of a current frame. The proposed method obtains a denoised pose using the Gaussian kernel obtained through this network to calculate a temporally weighted sum for an input noisy pose sequence. In [26], 3D human pose is represented in the form of 126 joint angles, and the weighted sum is computed for this joint angle sequence. Our work provides a more reliable basis for computation in non-Euclidean space where 3D rotation actually exists. While the values of weights are limited by the Gaussian kernel in [26], they are not in our method.

Several methods have been proposed to refine the SMPL pose parameter [9,10]. Kanazawa et al. [9] proposed a regressor that performs iterative refinement to estimate the SMPL parameter. Kolotouros et al. [10] presented a method that refines the estimated SMPL parameter through an optimization process. In [9,10], the refinement process is included in the model, which outputs SMPL identity and pose parameters directly from an input image. Our refinement method is independent of the pose estimation model and can be applied to the results of any method for estimating the SMPL pose parameter sequence regardless of their network structure. Our work is the first to propose a post-processing method for SMPL pose parameter refinement, and the proposed method is simple but works effectively.

## 3. Proposed Method

This section provides detailed descriptions of each module constituting our proposed HPR-Net. As presented in Section 1, we propose HPR-Net that generates a refined 3D human pose sequence from a noisy 3D human pose sequence estimated by other methods, such as VIBE. As shown in Figure 2, HPR-Net refines a noisy 3D pose of a target frame from input 3D poses that consist of all 3D poses within a window of size *N* (*N* is an odd number) centered on the target frame. We term these input 3D poses as a *pose chunk*. HPR-Net consists of a weight-regression module, a weighted-averaging module, and an SMPL module. Each module is explained in the following subsections. We first introduce the SMPL module to explain what 3D human model is used, how the 3D human pose is defined in the SMPL model, and why this module is needed in our framework. The weight-regression module consists of 1D convolution layers and generates an *N*-dimensional weight vector using non-local self-attention mechanism from an input pose chunk. The weighted-averaging module outputs a refined 3D pose by weighted averaging with the input pose chunk and weights from the weight-regression module. The above procedure is applied to the noisy 3D pose sequence with a sliding window manner, so we can obtain the refined 3D human pose sequence.

### 3.1. SMPL Module

SMPL is a 3D statistical shape model used to represent a human body and includes low-dimensional parameters to control the body shape. The parameter set included in the SMPL model consists of an identity parameter $\beta \in R^{10}$ and a pose parameter $\theta \in R^{72}$. The pose parameter represents the relative 3D rotations of 24 joints in an axis-angle form. This parameter controls the 3D pose of the human body represented by the SMPL model. From the given identity and pose parameters, the SMPL module generates a 3D human mesh model in a differentiable manner. The vertices $M\in R^{3\times 6890}$ of the generated mesh model are multiplied with a pretrained linear regression matrix included in the SMPL model, so that 24 joints $X_{smpl,3d}\in R^{3\times 24}$ can be additionally obtained. In HPR-Net, the SMPL module computes the human mesh model and the 3D joints from the refined pose parameters using our weighted-averaging module and the identity parameters estimated by existing methods. We can compare the 3D joints from the refined mesh generated by the SMPL module with its ground-truth to compute a loss function for learning and error for evaluating the proposed method. We also use the joint set $X_{3d}\in R^{3\times 14}$ obtained by converting $X_{smpl,3d}$ into 14 joints compatible with the joint definition of Human3.6M [27] for learning and evaluation.

### 3.2. Weight-Regression Module

**Figure 3:**
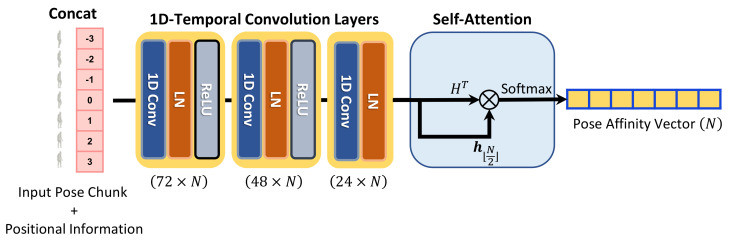
Detailed pipeline of the weight-regression module. ⊗ represents matrix multiplication. First, the weight-regression module concatenates positional information to an input pose chunk. Second, the positional encoded input chunk is fed into the weight-regression module that consists of three 1D temporal convolution layers. Finally, pose affinity vector is generated from the output temporal feature of the convolution layers.

**Network structure.** The weight-regression module of HPR-Net generates weights for pose refinement of the target pose from an input noisy pose chunk. Figure 3 shows the detailed structure of the weight-regression module consisting of 1D temporal convolution layer with a kernel size of 3, layer normalization [28], rectified linear unit activation, and self-attention layer. Suppose that $\Phi ={\left\{\beta_{i},\theta_{i}\right\}}_{i=0}^{N-1}$, which is a chunk of length *N* for the noisy SMPL parameter sequence, is given. Here, $\beta_{i}\in R^{10}$ and $\theta_{i}\in R^{72}$ are the identity and pose parameters in the *i*-th frame, respectively. The pose parameter $\theta_{i}$ represents the 3D rotations for 24 SMPL joints represented in an axis-angle form. We first convert $\theta_{i}$ to pose parameter $p_{i}\in R^{96}$ in a unit quaternion form. We apply frame-wise positional encoding to the unit quaternion pose chunk $P=\left[p_{0},\ldots ,p_{N-1}\right]\in R^{96\times N}$, similar to [12], before feeding it into the network. Specifically, to inject positional information into *P*, we concatenate a relative position index vector $\left[-\left\lfloor \frac{N}{2}\right\rfloor ,\ldots ,-1,0,1,\ldots ,\left\lfloor \frac{N}{2}\right\rfloor \right]$ with *P* to construct $\tilde{P}\in R^{97\times N}$ and feed the concatenated tensor into the weight-regression module. The weight-regression module first computes temporal feature $H=\left[h_{0},h_{1},\ldots ,h_{N-1}\right]\in R^{24\times N}$ from $\tilde{P}$ through three 1D temporal convolution layers. $h_{i}\in R^{24}$ represents the temporal feature of the *i*-th frame. A pose affinity vector $w\in R^{N}$ is generated through a non-local self-attention mechanism [12,13] as follows:

(1) $$w=\mathrm{Softmax}(H^{T}\cdot h_{\left\lfloor \frac{N}{2}\right\rfloor }).$$

Similar to the existing self-attention-based methods, our weight-regression module simply uses matrix multiplication and softmax operation to construct pose affinity vector $w={\left[w_{0},w_{1},\ldots ,w_{N-1}\right]}^{T}$, where $w_{i}\in R$ represents how the pose of *i*-th frame affects the computation of the refined pose at the $\left\lfloor \frac{N}{2}\right\rfloor$-th (i.e., center) frame. As our HPR-Net refines the center frame’s pose $p_{\left\lfloor \frac{N}{2}\right\rfloor }$ from *P*, we choose the center frame’s feature $h_{\left\lfloor \frac{N}{2}\right\rfloor }$ from *H* to compare the feature with all other features within the chunk.

**Why do we use LayerNorm?** From our experiments, we observed that the use of layer normalization after the convolution layer shows higher performance than the commonly used batch normalization [29]. In our method, the 3D pose in an input pose chunk consists of 3D rotations, and this 3D rotation is represented in a unit quaternion form that is geometrically on a 4D unit sphere. Layer normalization helps to learn the weight-regression module by enforcing the features extracted through the convolution layer to be on the unit sphere.

### 3.3. Weighted-Averaging Module

**Figure 4:**
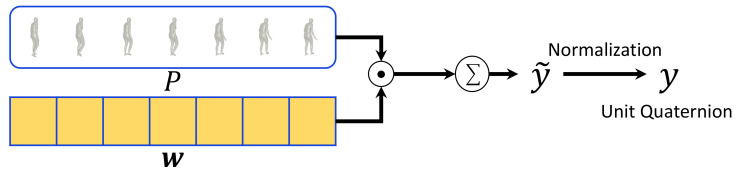
Detailed pipeline of the weighted-averaging module. ⊙ represents element-wise multiplication with broadcasting. Σ represents summation for across time dimension. Input pose vectors *P* are multiplied with pose affinity weights w which are generated by the weight-regression module. Then weighted pose vectors are added to output a refined pose vector $\tilde{y}$. To ensure that the refined pose parameters consist of unit quaternions, we additionally normalize $\tilde{y}$ to output a valid pose vector y.

**Pose refinement by weighted averaging.** Using w generated by the weight-regression module, we perform weighted averaging on the input pose chunk *P* and obtain the refined pose $y\in R^{96}$ as follows. Figure 4 shows the detailed structure of the weighted-averaging module. Weighted averaging cannot be directly applied to 3D rotations because they are defined in non-Euclidean space. Therefore, we obtain a second-order approximation of optimal rotation averaging by performing weighted averaging based on unit quaternion following Gramkow’s work [6]. By weighted averaging, we first obtain $\tilde{y}$ as follows:

(2) $$\tilde{y}=\sum_{i=0}^{N-1}w_{i}p_{i},$$

where $w_{i}$ is the *i*-th component of vector w and represents the contribution of $p_{i}$ to weighted averaging. However, $\tilde{y}={\left[{\tilde{q}}_{0}^{T},{\tilde{q}}_{1}^{T},\ldots ,{\tilde{q}}_{23}^{T}\right]}^{T}$ cannot be guaranteed to consist of unit quaternions. Therefore, we additionally perform normalization to make the 3D rotations ${\tilde{q}}_{j}$ belonging to $\tilde{y}$ into a unit quaternion form using $q_{j}={\tilde{q}}_{j}/∥{\tilde{q}}_{j}∥$. The weighted-averaging module outputs the refined 3D pose y consisting of unit quaternions as follows:

(3) $$y={\left[q_{0}^{T},q_{1}^{T},\ldots ,q_{23}^{T}\right]}^{T}\in R^{96},$$

where $q_{j}$ denotes the 3D rotation of the *j*-th SMPL joint.

**Loss functions.** The refined 3D human pose y is converted to an axis-angle form and then fed into the SMPL module along with the identity parameter β estimated by other methods to generate the refined mesh $\hat{M}$ and 3D joints ${\hat{X}}_{3d}=\left[{\hat{x}}_{3d,1},\ldots ,{\hat{x}}_{3d,14}\right]$. The joint loss function $L_{\mathrm{joint}}$ for learning the proposed network is defined as follows:

(4) $$L_{\mathrm{joint}}=\frac{1}{J}\sum_{j=1}^{J}{∥{\hat{x}}_{3d,j}-x_{3d,j}∥}_{1},$$

where J=14 is the number of joints, and ${\hat{x}}_{3d,j}$ and $x_{3d,j}$ denote the estimated and ground-truth coordinate vectors of the *j*-th joint, respectively. $L_{\mathrm{joint}}$ is defined as L1 loss and we supervise only ${\hat{X}}_{3d}$ that is generated from $\hat{M}$ by the SMPL module.

## 4. Experimental Results

### 4.1. Datasets and Evaluation Metrics

We use Human3.6M [27] and 3DPW [30] for training and evaluation. Human3.6M is a large-scale dataset obtained from an indoor environment, and has been used in many existing 3D human pose estimation methods. The Human3.6M dataset consists of videos, where 11 subjects perform 15 actions and includes 2D and 3D joint annotations for each frame. Image data in Human3.6M were captured in four camera views. 3DPW is a dataset obtained in an in-the-wild environment. The 3DPW dataset includes 60 videos and is divided into train, validation, and test sets. The three sets consist of 24, 12, and 24 videos, respectively. The 3DPW dataset provides 2D and 3D joint annotations and SMPL parameter annotations.

We split each dataset into training and test data. In Human3.6M, we use 5 subjects (1, 5, 6, 7, 8) as training data and 2 subjects (9, 11) as test data following the convention of previous studies [3,4,10]. For the 3DPW dataset, we use the train and validation sets as training data and the test set as test data. For convenience of training and evaluation, we apply VIBE to the training data of Human3.6M and 3DPW datasets and store the estimated SMPL parameters offline. The saved results are used as input for training the proposed network. We apply SPIN [10], VIBE [3], and MEVA [4] to the test data of each dataset, store the estimated SMPL parameters offline, and use them as input for evaluation. At the training stage, we train the proposed HPR-Net using all the training data of each dataset. We then evaluate the proposed method by applying HPR-Net to the test data of each dataset and report the performance quantitatively and qualitatively.

To evaluate the performance of the proposed method, we report MPJPE, PA-MPJPE, MPVE, and acceleration error. MPJPE and PA-MPJPE are metrics used to evaluate joint position error. MPJPE calculates the average 3D joint distance (mm). PA-MPJPE calculates the average 3D joint distance (mm) after performing Procrustes alignment [31] on the estimated and ground-truth joint sets. MPVE calculates the average position error (mm) of the vertices of the generated SMPL mesh. Acceleration error [2] is a metric for evaluating the temporal smoothness (mm/s ${}^{2}$) of the estimated pose sequence. Acceleration vectors are computed for the 3D joint sequence, and the acceleration error is calculated as the average difference between the estimated and ground-truth acceleration vectors.

### 4.2. Implementation Details

HPR-Net is trained end-to-end, and the input pose chunk in the training process is determined by random shuffling at each iteration. Zero padding is applied to the temporal 1D convolution of the weight-regression module. We set the length *N* of the input pose chunk for training the HPR-Net to 17 and calculate the loss function for the joints of the refined mesh corresponding to the center frame. We use Adam [32] as the optimizer of the network. The learning rate is set to $10^{-4}$. We do not decay the learning rate during training. The batch size and the number of epochs are set to 64 and 20, respectively. In each epoch, 1000 iterations are performed. The learning rate, batch size, and number of epochs are determined through simple greedy search using the validation set of 3DPW. Pytorch [33] was used to implement the proposed method, which was trained with a single Nvidia RTX3090 GPU. In the evaluation process, the input pose chunk is not randomly determined and is fed into the network in the order of the frames of the evaluation video. We refine the input video except for 16 frames (i.e., 8 frames each at the beginning and end of the video). The pose chunk of length 17 is fed into HPR-Net in a sliding window manner with stride 1.

### 4.3. Ablation Study

In ablation experiments, we report how the hyperparameters and component changes affect the performance of HPR-Net. We use VIBE’s pose sequence estimation result as our HPR-Net’s input chunk. We set the length of the input pose chunk to 17 in all experiments, except for the pose chunk length ablation experiment. In ablation experiments, HPR-Net is evaluated on 3DPW test set.

**Pose chunk length.** To determine the optimal length *N* of the pose chunk, we perform training using various lengths and analyze the results. Table 1 shows the performance in accordance with the length of the input chunk. HPR-Net shows the best performance with length 17, except for PA-MPJPE. Thus, we set the pose chunk length to 17.

**Various loss combinations.** In the proposed method, only 3D joints are supervised to train HPR-Net using the joint loss function in Equation (4). To justify this condition, we conduct an experiment to investigate how various combinations of loss functions affect the performance of HPR-Net. Specifically, we perform direct supervision with the joint loss function $L_{\mathrm{joint}}$ and losses that can be defined using the outputs of HPR-Net. The mesh loss function $L_{\mathrm{mesh}}$ and the pose loss function $L_{\mathrm{pose}}$ are additionally defined as follows:

(5) $$L_{\mathrm{mesh}}=\frac{1}{6890}\sum_{v=1}^{6890}{∥{\hat{m}}_{v}-m_{v}∥}_{1},$$

(6) $$L_{\mathrm{pose}}=\frac{1}{24}\sum_{j=1}^{24}{∥{\hat{R}}_{j}-R_{j}∥}_{F}^{2}.$$

The mesh loss function $L_{\mathrm{mesh}}$ is defined as L1 loss, where ${\hat{m}}_{v}$ and $m_{v}$ denote the estimated and ground-truth coordinate vectors for the *v*-th vertex, respectively. The pose loss function $L_{\mathrm{pose}}$ is for the pose parameters, including 3D rotations, where ${\hat{R}}_{j}$ and $R_{j}\in R^{3\times 3}$ denote the estimated and ground-truth rotation matrices for the *j*-th joint, respectively. Frobenius norm for their difference represents the distance (i.e., chordal distance [5]) between two 3D rotations in non-Euclidean space. The total loss function *L* for this ablation experiment is defined as follows:

(7) $$L=\lambda_{j}L_{\mathrm{joint}}+\lambda_{m}L_{\mathrm{mesh}}+\lambda_{p}L_{\mathrm{pose}},$$

where $\lambda_{j}$, $\lambda_{m}$, and $\lambda_{p}$ denote the weights that determine the strength of each loss.

Table 2 shows the performance of HPR-Net in accordance with the weights of *L*. HPR-Net shows the highest performance, except for PA-MPJPE when only the joint loss function $L_{\mathrm{joint}}$ is used. Using the pose loss function $L_{\mathrm{pose}}$ leads to performance degradation (1st, 4th, 6th, 7th rows). Supervising the mesh vertices shows lower PA-MPJPE (2nd row) than only using $L_{\mathrm{joint}}$ (3rd row). We use Human3.6M and 3DPW datasets for training. However, the Human3.6M dataset does not include SMPL annotations. Thus, only the 3DPW dataset is used to supervise the network when we calculate $L_{\mathrm{mesh}}$ and $L_{\mathrm{pose}}$. The size of 3DPW training data is smaller than that of Human3.6M. However, the experimental result from supervising with only $L_{\mathrm{mesh}}$ shows the highest PA-MPJPE and second highest performance on other metrics. If more datasets containing SMPL annotations are available, then the use of the mesh loss function will lead to further performance improvements.

**Positional encoding.** Most of the non-local attention-based methods inject positional information into their input. HPR-Net performs positional encoding, which helps to distinguish the pose of each frame in input pose chunk. We investigate the effect of positional encoding and its method on the performance of HPR-Net. Table 3 shows the performance of HPR-Net in accordance with the positional encoding method. For the experiment, we train and evaluate with three different models, one without positional encoding (*None*), one with sinusoidal positional encoding according to [12] (*Sinusoidal*), and one with positional encoding used in the proposed method (*Ours*). When positional encoding is not used, HPR-Net shows decreased PA-MPJPE performance compared with VIBE, but the other metrics are improved. Using the sinusoidal positional encoding shows improved results and best performance on PA-MPJPE. Our encoding method shows slightly lower PA-MPJPE compared with the sinusoidal positional encoding, but the best performance on the other metrics.

**Layer normalization.** The weight-regression module is composed of simple 1D temporal convolution layers. Layer normalization is adopted as the feature normalization layer of the proposed weight-regression module. To justify the use of layer normalization for HPR-Net, we trained three models, one without feature normalization, one using batch normalization, and one using layer normalization. Table 4 shows the performance comparison in accordance with the normalization method used in HPR-Net. When layer normalization is used, HPR-Net achieves the best performance in all metrics compared with other methods. From the result, layer normalization helps the learning of the weight-regression module.

### 4.4. Refinement on State-of-the-Art Methods

We evaluate the performance of applying HPR-Net to state-of-the-art methods [3,4,10] for different datasets [27,30]. Table 5 and Table 6 report the performance of existing methods and their refinement performance by HPR-Net on each evaluation dataset. Existing methods are re-evaluated using publicly provided pretrained models. HPR-Net achieves performance improvement in all metrics for all methods on 3DPW and Human3.6M datasets. HPR-Net considerably improves the acceleration error in every experiments. We trained our HPR-Net with the pose estimation result by VIBE as input. However, HPR-Net consistently improves other methods (i.e., SPIN and MEVA). These results show our HPR-Net’s generalization capability for other methods.

### 4.5. Comparison with Other Pose Refinement Methods

The pose parameter sequence can be refined in several methods. We compare HPR-Net with other methods in improving the pose sequence. Table 7 shows the quantitative improvement results of SLERP, Gaussian-filtering-based method (HPR-Gaussian), direct-regression-based method (HPR-DR), and HPR-Net. All the methods are evaluated on 3DPW test set. SLERP calculates the interpolated unit quaternion between two unit quaternions. MEVA uses SLERP to further smoothen their output pose parameter sequence. We test SLERP to evaluate its refinement performance and compare it with our HPR-Net’s performance. HPR-Gaussian regresses standard deviations to create optimal joint-wise Gaussian kernels. We implement the HPR-Gaussian model by modifying the structure of the weight-regression module in HPR-Net. We only change the kernel size of the 3rd temporal 1D convolution layer of the weight-regression module to *N* and set the number of channels to 24. HPR-Gaussian’s weight-regression module creates 24 joint-wise standard deviations, where the 24 Gaussian kernels with kernel size *N* are created. Each kernel is used for Gaussian filtering for the 3D rotation of each of the 24 joints. Specifically, weighted averaging of 3D rotations along the temporal axis is performed using the values of the kernels as weights. HPR-DR directly regresses the refined pose of the center frame from the input pose chunk. To implement HPR-DR, our proposed HPR-Net is modified as follows. We change the number of channels and kernel size in the last 1D convolution layer of the weight-regression module to 96 and *N*, respectively, so that the modified network (i.e., HPR-DR) generates a 96D vector. This vector is converted into a refined pose vector consisting of unit quaternions through normalization.

From the quantitative improvement results of each method in Table 7, we observe that SLERP does not improve the performance, except for the acceleration error. Acceleration is defined as the second derivative of the joint position and is very sensitive to the small noise in the refined pose sequence. Since SLERP performs weighted averaging for interpolation between two poses, it is effective in reducing the small noise and the acceleration error. HPR-Gaussian improves VIBE quantitatively. However, the performance gain for the acceleration error is smaller than SLERP because HPR-Gaussian over-smooths the pose sequence. HPR-DR fails to refine the results of VIBE. It is because the size of the training data, which is not large enough to train the direct regression model, leads to overfitting. Our HPR-Net adaptively adjusts the shape of the kernel to prevent over-smoothing and outperforms the other methods in all metrics, especially the acceleration error. Experimental results show that our HPR-Net is superior to the other human pose refinement methods.

### 4.6. Network Design Based on Non-Local Attention

**Figure 5:**
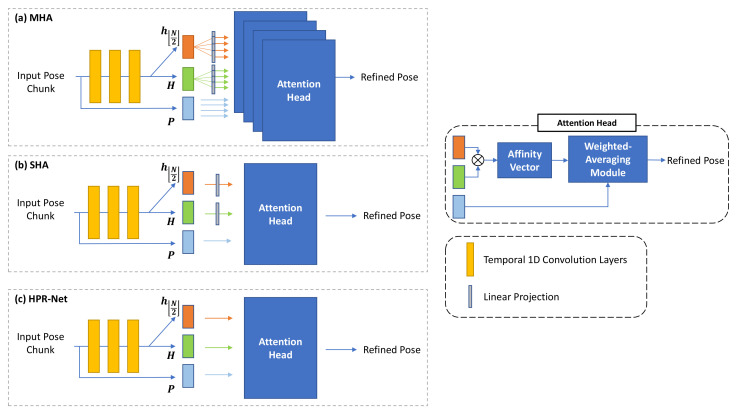
Detailed pipelines of multi-head structure (**a**), linear projection structure (**b**), and our proposed HPR-Net’s structure (**c**) for network design experiment. We did not apply linear projection to input pose chunk *P* in (**a**–**c**), because it should be averaged with affinity weights. Attention head contains affinity vector generation by self-attention and weighted-averaging processes.

Our proposed HPR-Net is based on non-local attention. Transformer [12] is a representative method and has the non-local attention-based structure. Our HPR-Net’s network structure is similar to that of the Transformer’s non-local self-attention module, but HPR-Net does not include components, such as multi-head attention and linear projection. To explore how these components affect our model, we compare our HPR-Net with two HPR-Net variants with a multi-head attention structure (MHA) and a single-head attention structure (SHA). The details of each structure are shown in Figure 5.

Table 8 shows the VIBE refinement performance of MHA, SHA, and our HPR-Net on the 3DPW dataset. All the experimented structures show acceleration error improvement. MHA shows higher MPJPE, PA-MPJPE, and MPVE than SHA. However, the two models fail to improve MPJPE, PA-MPJPE, and MPVE compared with VIBE. Unlike the two structures, HPR-Net improves the performance in all metrics and achieves the lowest acceleration error. The difference between our method and the two structures are that the features obtained from the convolution layers are not linearly projected, and the MHA is not used in the proposed HPR-Net. From the results, the linear projection layer seems to cause performance degradation by confusing to generate an appropriate affinity weight vector from input pose information. The MHA seems to result in overfitting by complicating the network structure more than necessary. Our network has a simpler structure and performs better. HPR-Net is more optimal in solving our problem than the commonly used non-local attention structure.

### 4.7. Qualitative Results

**Figure 6:**
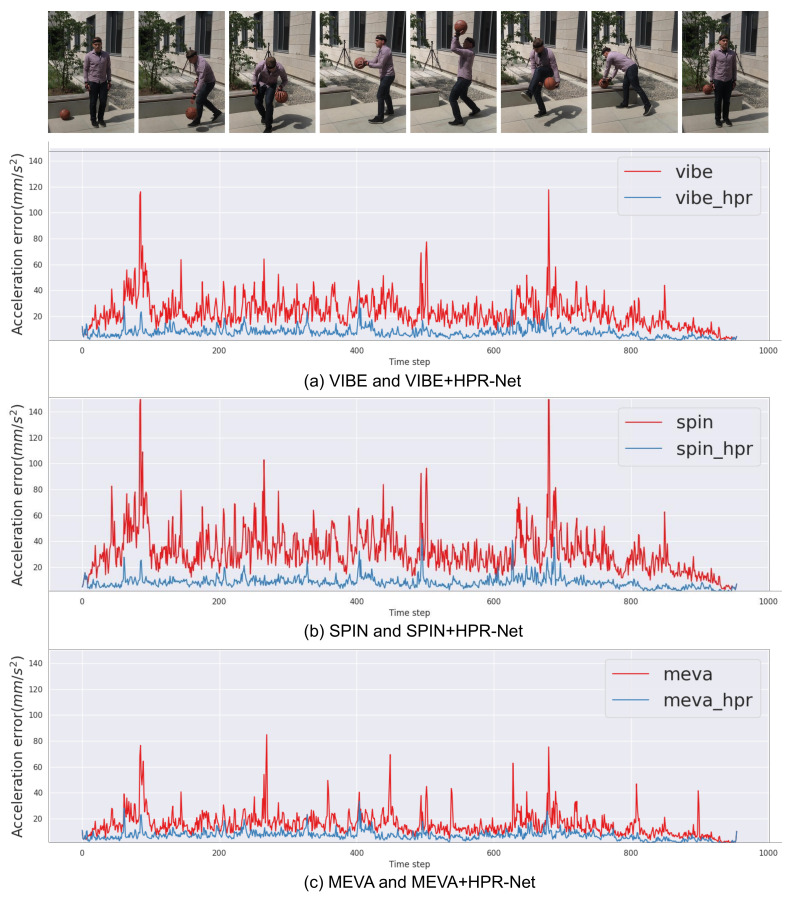
Comparison of acceleration error between HPR-Net and previous methods (VIBE, SPIN, and MEVA). HPR-Net effectively suppresses acceleration error for all methods, even there are very high peaks of acceleration error.

**Acceleration error improvement.** HPR-Net consistently shows a significant improvement in acceleration error across all methods and datasets on the basis of quantitative results. We present qualitative improvement results using a graph. Figure 6 shows the acceleration error of VIBE, SPIN, MEVA and their refined results after applying HPR-Net to each method. The acceleration errors are calculated for every three consecutive frames from a video of 3DPW. Compared with existing methods’ result, HPR-Net effectively improves the acceleration errors for all methods. In particular, the acceleration error is significantly reduced in frames with high peaks where the errors are noticeable.

**Figure 7:**
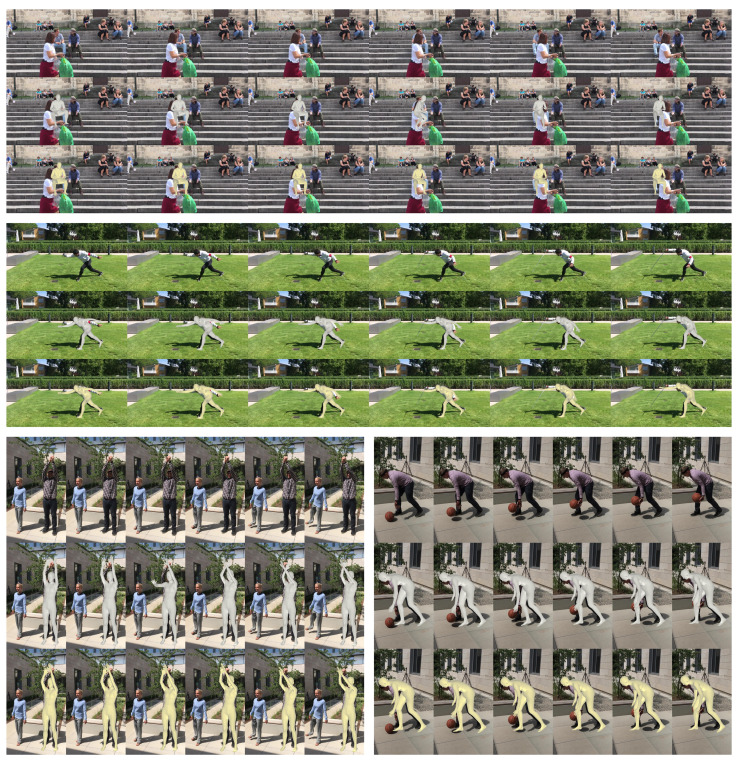
Input images (**top**) and reconstruction results of VIBE (**middle**, gray SMPL mesh) and HPR-Net (**bottom**, yellow SMPL mesh) on the 3DPW dataset.

**Figure 8:**
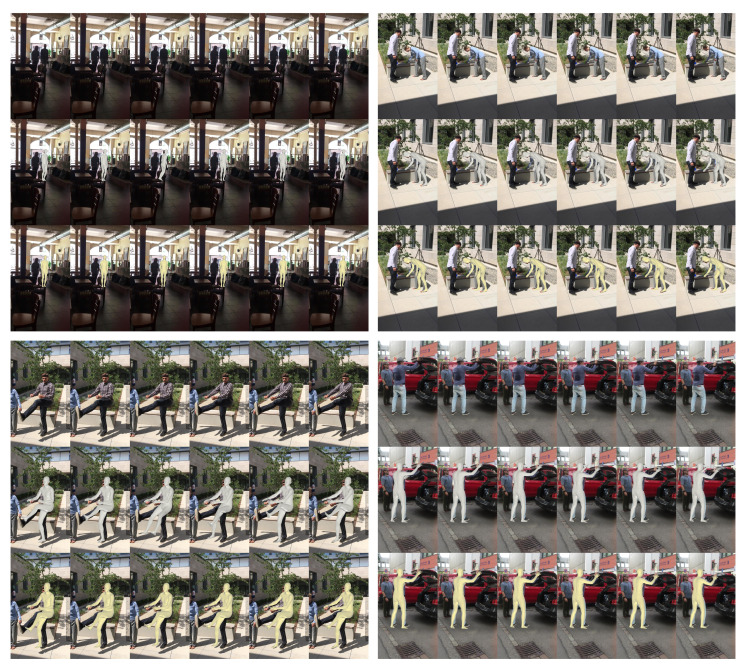
Input images (**top**) and reconstruction results of SPIN (**middle**, gray SMPL mesh) and HPR-Net (**bottom**, yellow SMPL mesh) on the 3DPW dataset.

**Refinement result.** We present the qualitative results to show that HPR-Net substantially refines a 3D human pose sequence estimated by existing methods. Figure 7 and Figure 8 show the refined results for VIBE and SPIN, respectively. For each example in Figure 7 and Figure 8, the top, middle, and bottom rows show the input image sequence, the estimation result by the existing method, and the refinement result by the proposed HPR-Net, respectively. We do not report the qualitative result for MEVA, because the SMPL estimation results by MEVA’s official code are projected incorrectly in the image. In the topmost example of Figure 7, a pedestrian causes occlusion. Thus, the pose of the target subject is incorrectly estimated. HPR-Net refines the results by reconstructing the appropriate pose using the information of nearby frames. In the top-left example of Figure 8, SPIN predicts the global orientation incorrectly due to challenging illumination. This incorrect global orientation is well refined in the result of HPR-Net. From the other results, HPR-Net refines the incorrect estimations of arms and legs.

### 4.8. Discussion

In accordance with our experimental results, the refinement of the human pose sequence estimated by existing methods can be achieved through a data-driven approach on the basis of a large-scale dataset and a deep neural network. To realize this, the proposed HPR-Net adaptively performs weighted averaging, a well-known framework for noise reduction, on input data, therefore consistently improving the human pose estimation performance of existing state-of-the-art methods. The pose refinement by HPR-Net is performed independently of the existing human pose estimation method. This modularity can be a benefit of our approach because it makes the use and analysis of the proposed method easy. However, HPR-Net has a limitation of depending on the pose estimation results of existing methods. Combining HPR-Net with the existing pose estimation network and learning it in an end-to-end manner may bring additional performance improvement. We plan to continue our research to investigate the end-to-end approach and overcome the limitations of the proposed method.

## 5. Conclusions

We propose HPR-Net to refine the noisy 3D human pose parameter sequence. HPR-Net improves the accuracy and temporal smoothness of the 3D human pose sequence through a simple non-local attention-based weighted averaging for a noisy pose parameter chunk represented in a unit-quaternion form. We report quantitatively and qualitatively that the proposed method can improve 3D human reconstruction performance for various real datasets, such as Human3.6M and 3DPW. From the experiments for improving the results of various existing methods such as SPIN, VIBE, and MEVA, a consistent performance improvement is observed regardless of the method used to estimate the input human pose sequence. This finding shows that our method works in a model-agnostic manner. The superiority of HPR-Net is confirmed by comparing it with other approaches that can refine 3D human pose parameters.

## Figures and Tables

**Table 1:** Performance comparison of HPR-Net according to different pose chunk length. Bold values indicate best results.

Length	MPJPE ↓	PA-MPJPE ↓	MPVE ↓	Accel-Error ↓
9	82.14	51.82	98.25	7.31
17	**81.10**	51.26	**97.13**	**6.94**
33	82.11	51.63	98.26	18.36
65	81.23	**50.97**	97.24	8.19
129	81.81	51.35	97.89	11.69

**Table 2:** Performance comparison of HPR-Net according to various combinations of loss functions. (✓ = 1.0, blank = 0.0). Bold values indicate best results.

$\lambda_{j}$	$\lambda_{m}$	$\lambda_{p}$	MPJPE ↓	PA-MPJPE ↓	MPVE ↓	Accel-Error ↓
		✓	85.60	55.03	102.07	12.39
	✓		81.29	**51.04**	97.33	7.72
✓			**81.10**	51.26	**97.13**	**6.94**
✓		✓	84.37	54.12	100.76	10.38
✓	✓		81.46	51.16	97.48	9.79
	✓	✓	85.64	55.20	102.12	12.11
✓	✓	✓	83.76	53.39	100.06	14.12

**Table 3:** Comparison of refinement performance of HPR-Net according to positional encoding method. Bold values indicate best results.

Methods	MPJPE ↓	PA-MPJPE ↓	MPVE ↓	Accel-Error ↓
None	81.63	52.00	97.72	6.97
Sinusoidal	81.53	**51.15**	97.58	8.42
Ours	**81.10**	51.26	**97.13**	**6.94**

**Table 4:** Comparison of refinement performance of HPR-Net according to feature normalization method. Bold values indicate best results.

Methods	MPJPE ↓	PA-MPJPE ↓	MPVE ↓	Accel-Error ↓
None	82.12	51.84	98.17	7.76
BatchNorm	82.66	52.07	98.81	12.93
LayerNorm	**81.10**	**51.26**	**97.13**	**6.94**

**Table 5:** HPR-Net’s pose refinement performance for various existing methods on 3DPW test data. Bold values indicate performance improvements.

Methods	MPJPE ↓	PA-MPJPE ↓	MPVE ↓	Accel-Error ↓
VIBE	82.28	51.72	98.42	20.69
VIBE + HPR-Net	**81.10**	**51.26**	**97.13**	**6.94**
SPIN	102.46	60.05	129.22	29.78
SPIN + HPR-Net	**100.95**	**59.30**	**127.58**	**8.19**
MEVA	85.81	53.54	102.18	14.37
MEVA + HPR-Net	**85.43**	**53.50**	**101.79**	**6.63**

**Table 6:** HPR-Net’s pose refinement performance for various existing methods on Human3.6M test data. Bold values indicate performance improvements.

Methods	MPJPE ↓	PA-MPJPE ↓	Accel-Error ↓
VIBE	78.35	53.58	9.76
VIBE + HPR-Net	**77.77**	**53.17**	**2.13**
SPIN	68.22	46.16	14.21
SPIN + HPR-Net	**67.35**	**45.53**	**2.74**
MEVA	73.64	48.48	7.22
MEVA + HPR-Net	**73.06**	**48.06**	**1.83**

**Table 7:** Comparison of refinement performance between HPR-Net and other pose sequence refinement methods on the 3DPW dataset. Bold values indicate best results.

Methods	MPJPE ↓	PA-MPJPE ↓	MPVE ↓	Accel-Error ↓
SLERP	82.72	52.13	99.88	12.38
HPR-Gaussian	82.15	51.58	98.30	18.04
HPR-DR	183.01	102.79	223.20	14.28
HPR-Net	**81.10**	**51.26**	**97.13**	**6.94**

**Table 8:** Comparison of refinement performance according to network design of HPR-Net. Bold values indicate best results.

Methods	MPJPE ↓	PA-MPJPE ↓	MPVE ↓	Accel-Error ↓
MHA	84.00	53.20	99.94	7.71
SHA	84.13	53.52	100.44	7.49
HPR-Net	**81.10**	**51.26**	**97.13**	**6.94**
